# Multi-centre, randomised non-inferiority trial of early treatment versus expectant management of patent ductus arteriosus in preterm infants (the BeNeDuctus trial): statistical analysis plan

**DOI:** 10.1186/s13063-021-05594-x

**Published:** 2021-09-15

**Authors:** Tim Hundscheid, Rogier Donders, Wes Onland, Elisabeth M. W. Kooi, Daniel C. Vijlbrief, Willem B. de Vries, Debbie H. G. M. Nuytemans, Bart van Overmeire, Antonius L. Mulder, Willem P. de Boode, on behalf of the BeNeDuctus trial study group, Peter H. Dijk, Anton H. L. C. van Kaam, Tessa de Baat, Koen P. Dijkman, Eduardo Villamor, André A. Kroon, Remco Visser, Susanne M. de Tollenaer, Filip Cools, Marisse Meeus, Anne-Britt Johansson, Frank Derriks, Catheline Hocq, Alexandra Zecic, Tine Brink Henriksen, Kasper Jacobsen Kyng

**Affiliations:** 1 Radboud Institute for Health Sciences, Amalia Children’s Hospital, Department of Paediatrics, Division of Neonatology, Radboud University Medical Centre Nijmegen, Internal postal code 804, Geert Grooteplein Zuid 10, 6525 GA Nijmegen, The Netherlands; 2 Department for Health Evidence, Radboud University Medical Centre, Geert Grooteplein Zuid 10, 6525 GA Nijmegen, The Netherlands; 3 Emma Children’s Hospital Amsterdam University Medical Centers, Department of Neonatology, University of Amsterdam, Vrije Universiteit Amsterdam, Meibergdreef 9, 1105 AZ Amsterdam, The Netherlands; 4 Beatrix Children’s Hospital, Department of Paediatrics, Division of Neonatology, University Medical Center Groningen, Hanzeplein 1, 9713 GZ Groningen, The Netherlands; 5 Utrecht University, Wilhelmina Children’s Hospital, Division of Woman and Baby, Department of Neonatology, University Medical Centre Utrecht, Lundlaan 6, 3584 EA Utrecht, The Netherlands; 6 Wilhelmina Children’s Hospital, Division of Woman and Baby, Department of Neonatology, University Medical Centre Utrecht, Utrecht University, Lundlaan 6, 3584 EA Utrecht, The Netherlands; 7 Neonatology Network Netherlands (N3), p/a Meibergdreef 9, 1105 AZ Amsterdam, The Netherlands; 8 Opgroeien, Flemish Government, Brussels, Belgium; 9 Department of Paediatrics, Division of Neonatology, Antwerp University Hospital, Wilrijkstraat 10, 2650 Edegem, Belgium; 10 Radboud Institute for Health Sciences, Amalia Children’s Hospital, Department of Paediatrics, Division of Neonatology, Radboud University Medical Centre, Geert Grooteplein Zuid 10, 6525 GA Nijmegen, The Netherlands

**Keywords:** Prematurity, Patent ductus arteriosus, Neonatal intensive care unit, Ibuprofen, Expectant management, Ductal ligation, Mortality, Necrotising enterocolitis, Bronchopulmonary dysplasia, Statistical analysis plan

## Abstract

**Background:**

Controversy exists about the optimal management of a patent ductus arteriosus (PDA) in preterm infants. A persistent PDA is associated with neonatal mortality and morbidity, but causality remains unproven. Although both pharmacological and/or surgical treatment are effective in PDA closure, this has not resulted in an improved neonatal outcome. In most preterm infants, a PDA will eventually close spontaneously, hence PDA treatment potentially increases the risk of iatrogenic adverse effects. Therefore, expectant management is gaining interest, even in the absence of convincing evidence to support this strategy.

**Methods/design:**

The BeNeDuctus trial is a multicentre, randomised, non-inferiority trial assessing early pharmacological treatment (24–72 h postnatal age) with ibuprofen versus expectant management of PDA in preterm infants in Europe. Preterm infants with a gestational age of less than 28 weeks and an echocardiographic-confirmed PDA with a transductal diameter of > 1.5 mm are randomly allocated to early pharmacological treatment with ibuprofen or expectant management after parental informed consent.

The primary outcome measure is the composite outcome of mortality, and/or necrotizing enterocolitis Bell stage ≥ IIa, and/or bronchopulmonary dysplasia, all established at a postmenstrual age of 36 weeks. Secondary short-term outcomes are comorbidity and adverse events assessed during hospitalization and long-term neurodevelopmental outcome assessed at a corrected age of 2 years. This statistical analysis plan focusses on the short-term outcome and is written and submitted without knowledge of the data.

**Trial registration:**

ClinicalTrials.gov NTR5479. Registered on October 19, 2015, with the Dutch Trial Registry, sponsored by the United States National Library of Medicine Clinicaltrials.gov NCT02884219 (registered May 2016) and the European Clinical Trials Database EudraCT 2017-001376-28.

## Background

A patent ductus arteriosus (PDA) is common in preterm infants [1] and is associated with neonatal mortality and morbidity, such as bronchopulmonary dysplasia (BPD), necrotizing enterocolitis (NEC) and intraventricular haemorrhage (IVH). Controversy exists about its optimal management, as early PDA treatment induces PDA closure but does not improve overall outcome [2–4].

Ductus arteriosus closure is delayed in prematurity, since it is not yet programmed for prompt postnatal closure [5]. This is supported by the observation of a high amount of spontaneous closure [6] even after ‘failed’ pharmacological treatment [7]. Since early pharmacological treatment has not been proven to improve outcome, it potentially increases the risk of iatrogenic adverse effect in patients in whom the PDA would have closed spontaneously. These observations have led to an increased interest in expectant PDA management [8]. Evidence to support expectant PDA management is scarce and conflicting [9], due to a high amount of open label treatment in placebo-controlled randomised controlled trials (RCTs) [4, 10] and a heterogeneous definition of (haemodynamic significant) PDA [11].

The BeNeDuctus trial assesses early pharmacological treatment within 24–72 h postnatal age (PNA) with ibuprofen versus expectant management of PDA in preterm infants in Europe. The study protocol was published previously [10]. This paper describes the statistical analysis plan (SAP) for short-term outcomes in detail, which is written and submitted without knowledge of the data.

### Objectives

The primary aim of the BeNeDuctus trial is to investigate whether an expectant management is non-inferior to early treatment (24–72 h PNA) for PDA (diameter > 1.5mm) in preterm infants, born at a gestational age less than 28 weeks, with regard to the composite outcome of mortality and/or NEC (Bell stage ≥ IIa) and/or BPD at a postmenstrual age (PMA) of 36 weeks.

## Methods/design

### Design and setting

The BeNeDuctus trial is a multicentre, non-inferiority trial in preterm infants with an echocardiographic-confirmed PDA > 1.5 mm before 72 h of PNA. Patients are recruited from 17 neonatal intensive care units (NICUs) in the Netherlands, Belgium and Denmark.

### Study protocol development and conduct

The BeNeDuctus trial is registered with the Dutch Trial Register NTR5479 (registered on 19 October 2015), the registry sponsored by the United States National Library of Medicine Clinicaltrials.gov NCT02884219 (registered May 2016) and the European Clinical Trials Database EudraCT 2017-001376-28. This study has been approved by the medical ethics committee of the Radboud University (CMO Arnhem-Nijmegen; Number 2016-2552/NL57885.091.16). The local ethics committee of each participating hospital approved the local feasibility of the study protocol.

During the course of the study the ethics committee approved two amendments. On 25 September 2017, the study protocol was adapted for use by participating sites outside The Netherlands, the data monitoring plan was updated and cyclo-oxygenase inhibitor (i.e. indomethacin and/or ibuprofen) was changed to ibuprofen due to unavailability of indomethacin. On 26 February 2018, reimbursement of investigational medicinal product in Belgium was added and the Data Safety Monitoring Board (DSMB) paragraph was edited according to an updated DSMB charter.

The trial is conducted according to the principles of the Declaration of Helsinki [12], good clinical practice guidelines and Dutch, Belgian and Danish legislation regarding medical research involving human subjects [13]. Informed consent from both parents needs to be obtained before patients can be randomised in the trial.

An independent DSMB monitors the progress of the study and performs interim-analysis on safety. The trial will be reported according to the Consolidated Standards of Reporting Trials (CONSORT) guidelines [14].

In- and exclusion criteria are described in detail in the previously published study protocol [10]. In summary, preterm infants born at a gestational age of less than 28 weeks with an echocardiographic-confirmed PDA with a diameter > 1.5 mm and transductal (predominantly) left-to-right shunting at a PNA of less than 72 h are eligible.

Included patients are randomised to either early pharmacological treatment with ibuprofen, dosage according to local protocol or expectant management. The use of ‘open-label’ pharmacological treatment in the expectant management group is most definitely discouraged and only allowed when predefined open-label treatment criteria are met [10]. In a strict sense open-label treatment is a misnomer, since in this non-placebo controlled nor blinded clinical trial, no label could be opened in the expectant management study group. Due to the widespread use of the term open label in PDA research, we will continue to use this term for pharmacological treatment in the expectant treatment group.

### Randomisation and data collection

Eligible patients are randomly allocated to early pharmacological treatment or expectant management per centre and stratified according to gestational age in two strata (stratum A less than 26^0/7^ weeks’ gestation or stratum B 26^0/7^–27^6/7^ weeks’ gestation). Stratification per centre was done for pragmatic reasons and is not likely to influence the primary outcome. Therefore, the analyses will not be adjusted for centre. In an additional analysis, the primary outcome will be adjusted for gestational age, as this is a prognostic covariate, using Poisson regression. Block size varies from four to eight. Multiple birth infants are preferably randomised independently, unless there is an explicit request from the parents to expose all siblings to the same treatment group as the first patient is allocated to. The randomisation process is centrally controlled and web-based.

Data management is implemented according to good clinical practice guidelines. Patient data, both until hospital discharge and long-term follow-up data, are entered via an electronic case record form in a central web-based Castor® Database to facilitate on-site data entry [15]. The most important data is shown in Table 1 for baseline characteristics and Table 2 for outcome measures.

**Table 1:** Baseline maternal and patient characteristics of randomised patients

		Early pharmacological treatment (*n*= )	Expectant management (*n*= )
**Maternal characteristics**	Maternal age [years]		
	Maternal obstetrical disease		
	*Pre-eclampsia*		
	*HELLP syndrome*		
	*Placental abruption*		
	*PPROM*		
	*Clinical chorioamnionitis*		
	Maternal medication		
	*NSAIDs*		
	*Magnesium sulphate*		
	Tocolysis		
	Administration of ACS		
	*ACS course completed*		
	Type of delivery		
	*Vaginal*		
	*Caesarean section*		
	Multiple birth		
**Patient characteristics**	Gestational age [weeks]		
	Birth weight [grams]		
	Outborn (yes)		
	Gender (male)		
	Apgar score at 5 minutes		
	Resuscitation after birth		
	*Non-invasive respiratory support*		
	*Invasive respiratory support*		
	*Circulatory support*		
	Respiratory distress syndrome		
	Surfactant administration		
	PDA diameter [mm]		

Data is presented as mean (± standard deviation) or median [interquartile ranges]. Categorical variables are presented as counts (percentage)

*ACS* antenatal corticosteroids, *HELLP* haemolysis, elevated liver enzymes and low platelets, *PDA* patent ductus arteriosus, *PPROM* preterm premature rupture of membranes, *NSAIDs* non-steroidal anti-inflammatory drugs, *TTN* transient tachypnea of the newborn

**Table 2:** Outcome parameters per analysis

		Intention-to-treat		Per-protocol	
		Early R_x_ (*n* = )	Expectant (*n* = )	Early R_x_ (*n* = )	Expectant (*n* = )
***Primary composite outcome***					
	Mortality, BPD and/or NEC at 36 weeks PMA				
***Secondary outcomes***					
Treatment	Total doses of COXi				
	Surgical PDA ligation				
	Total fluid intake DOL 7 [ml/kg/day]				
	Co-interventions				
	*Postnatal steroids*				
	*Paracetamol use*				
	*Diuretics*				
Death	Mortality at 28 days PNA				
	Mortality at 36 weeks PMA				
	Mortality				
Haemodynamic	Cardiovascular support				
	*Volume expansion*				
	*Inotropes/vasopressors*				
	*Corticosteroids*				
	Hyperlactataemia				
	Renal failure				
	Hypertension				
Pulmonary	BPD at 28 days PNA				
	BPD at 36 weeks PMA				
	Supplemental oxygen [days]				
	Respiratory support [days]				
	*Invasive*				
	*Non-invasive*				
	Pulmonary haemorrhage				
	Pulmonary air leakage				
CNS	PVE				
	IVH				
	*Grade I-II*				
	*Grade ≥ III*				
	PHVD				
	Seizures				
GI	NEC (Bell stage ≥ IIa)				
	Gastrointestinal haemorrhage				
	SIP				
	Time to full enteral feeding [days]				
Infection	Sepsis				
	Meningitis				
	Pneumonia				
	Thrombocytopenia				
	Hyperglycaemia				
Eye	ROP				
	PLUS disease				
	ROP treatment				
Miscellaneous	Biometry at PMA 36 weeks				
	*Weigth [grams]*				
	*Length [cm]*				
	Biometry at discharge				
	*Weigth [grams]*				
	*Length [cm]*				
	Length of hospitalisation [days]				
	DA closed at discharge				

All outcome measures are till hospital discharge to home unless otherwise specified. Data is presented as mean (± standard deviation) or median [interquartile ranges]. Categorical variables are presented as counts (percentage)

*BPD* bronchopulmonary dysplasia, *CNS* central nervous system, *COXi* cyclo-oxygenase inhibitor, *DA* ductus arteriosus, *DOL* day of life, *GI* gastrointestinal, *IVH* intraventricular haemorrhage, *LF* low-flow, *MV* mechanical ventilation, *NEC* necrotizing enterocolitis, *PDA* patent ductus arteriosus, *PHVD* posthemorrhagic ventricular dilatation, *PMA* postmenstrual age, *PNA* postnatal age, *PVE* periventricular echogenicity, *ROP* retinopathy of prematurity, *R*_*x*_ pharmacological treatment, *SIP* spontaneous gastrointestinal perforation

### Primary outcome

The primary outcome is the dichotomous composite outcome of mortality, and/or NEC (Bell stage ≥ IIa) [16], and/or BPD at a PMA of 36 weeks. BPD is defined as the need for supplemental oxygen or positive pressure ventilatory support at a PMA of 36 weeks and diagnosed following international standard criteria by Bancalari [17], including an oxygen reduction test according to Walsh [18] (Table 2).

### Short-term secondary outcomes

Secondary outcomes are adverse events till discharge, which are shown in Table 2.

### Short-term secondary outcome definitions

Hyperlactataemia is defined as a serum concentration > 2.5 mmol/L, either capillary or arterial [19]. Renal failure is defined as creatinine > 120 μmol/L or urine output < 0.5 ml/kg/h. Hypertension is defined as mean arterial blood pressure > 95th percentile, according to Zubrow or Dionne [20, 21].

Respiratory support is divided in invasive, encompassing conventional and high-frequency ventilation, and non-invasive, encompassing non-invasive positive pressure ventilation, nasal continuous positive airway pressure and high-flow nasal cannula therapy. High-flow nasal cannula therapy is defined as a flow > 1 l per minute. Low flow, defined as flow < 1 l per minute, is considered as respiratory support for supplemental oxygen. Pulmonary air leakage is defined as pneumothorax, pulmonary interstitial emphysema or pneumomediastinum.

IVH is graded according to Volpe [22]. Periventricular echogenicity is graded according to Hashimoto [23].

Time to full enteral feeding is defined as the first moment full enteral feeding is reached, even in cases in which enteral feeding needs to be stopped afterwards, for example due to NEC.

Sepsis is defined as positive blood culture and need for antibiotics. Meningitis is defined as sepsis with antibiotic treatment regimen (dosage and duration) for meningitis, irrespective of spinal fluid culture. Thrombocytopenia is defined as a platelet count < 100 x 10^9^/L. Hyperglycaemia is defined as elevated glucose level necessitating insulin treatment according to local protocol.

Retinopathy of prematurity (ROP) is graded according to the international classification [24].

### Long-term secondary outcomes

Neurodevelopmental outcome is assessed in all Dutch and Belgian children in the National Neonatal Follow Up Program at a corrected age of 24 months by (a) paediatric and neurologic examination; (b) cognitive assessment with Bayley Scales of Infant and Toddler Development, Third Dutch Edition (BSID-III-NL); (c) behavioural assessment with Child Behaviour Check List (CBCL), Teacher Report Form (TRF) questionnaire. Despite the Movement Assessment Battery for Children, Second Dutch Edition (Movement ABC 2-NL) was mentioned in the study protocol, this will not be included in the neurodevelopmental outcome at 24 months corrected age as it is assessed at five years corrected age. Motor scales of the BSID-III-NL will be used to assess motor function. Equivalent assessments may be used for non-Dutch or Belgian children.

### Long-term secondary outcome definitions

Participants who are alive and have a BSID III cognitive and motor composite score at 2 years corrected age ≥ 85 (−1 standard deviation) will be defined as having survived without neurodevelopmental impairment.

General health is considered (a) good, i.e. no readmissions in first 2 years after discharge home; (b) moderate, i.e. 1–3 readmissions; or (c) poor, i.e. >3 readmissions.

Neurologic examination is subdivided in (a) normal, i.e. no symptoms of pathology; (b) mild abnormal, i.e. mild muscle tone abnormalities and coordination problems that do not hamper functioning and/or development; and (c) abnormal, i.e. muscle tone abnormalities and/or coordination problems that are hampering development, cerebral palsy and/or infantile encephalopathy.

Vision is scored as (a) normal, i.e. no problems with sight; (b) mildly abnormal, i.e. treated by ophthalmologist/orthoptist for abbreviation (goggles) or strabismus/amblyopia; (c) abnormal, i.e. limited vision, but ability to see anything; and (d) severely abnormal, i.e. blind.

Hearing is scored as (a) normal, i.e. no hearing problems; (b) mildly abnormal, i.e. light hearing loss for which control or treatment; (c) abnormal, i.e. neurosensory hearing loss (partially) corrected with hearing aid; and (d) severely abnormal, i.e. neurosensory hearing loss, deafness.

### Adverse events (AEs) and serious adverse events (SAE)

As this trial is undertaken in a fragile patient group, context-specific (S)AEs are not individually reported to the medical ethics committee, as described in the study protocol [10, 25, 26]. Non-context specific (S)AEs are reported to the medical ethics committee and will be listed.

### Statistical methods specified in the study protocol

### Sample size calculation

As described in the study protocol, the a priori risk of mortality, NEC and BPD is estimated to be 20%, 10% and 15%, respectively, for preterm infants born at a gestational age of less than 28 weeks, based on data from The Netherlands Perinatal Registry (PRN foundation) in the period 2008–2012. Non-inferiority is defined as a significant difference in the primary outcome parameter between the two groups of less than 10%. In other words, the one-side 95% confidence interval of the observed difference between an expectative approach and ibuprofen treatment should not exceed the non-inferiority margin of 10%.

With an estimated a priori risk for the composite of mortality and/or NEC and/or BPD at 36 weeks PMA of 35%, a one-sided type I error of 5% and a power of 80%, the sample size to exclude a non-inferiority margin of 10% for the difference of proportion of participants reaching the primary outcome parameter is 564 patients, being 282 patients in each group. This sample size is calculated using PASS 2008, version 08.0.8 NCSS.

### Original proposed analyses

The originally proposed analyses are described in the published study protocol [10]. For the primary outcome, a 95% one-sided confidence interval for the risk difference will be calculated, and when based on this interval, a difference of 10% or more can be excluded, non-inferiority will be concluded.

Treatment effects for the dichotomous clinical outcomes will be reported using risk differences with 95% confidence interval. Normally distributed data will be presented as mean ± standard deviations, uneven distributed data as medians with interquartile ranges. Categorical data will be analysed using the chi-square for two and multiway tables. Continuous data will be analysed using the Student’s *t* test. Both intention-to-treat and per-protocol analyses will be employed. Statistical significance is defined as a *p* value < 0.05. Here, we present our final and further detailed SAP.

### Interim analyses and safety reporting

To protect patients and to assist and advise the principal investigator in protecting the safety, validity and credibility of the trial, a DSMB is installed. The DSMB consists of a neonatologist, paediatric cardiologist and biostatistician. The members have no competing interests and are not involved in the trial. Their composition, tasks, responsibilities and working procedures are described in a charter (available on request).

Safety analysis is scheduled when 15%, 30%, 50% and 75% of the data are gathered. Before every interim analysis the DSMB receives a report from the trial coordinator and data manager, that includes data on (1) context-specific (S)AEs, subdivided in (a) hemodynamic, (b) pulmonary, (c) central nervous system, (d) gastrointestinal, (e) infectious, (f) renal, (g) ophthalmologic, (h) metabolic and (i) mortality; (2) non-context-specific (S)AEs; and (3) open-label treatment in expectant study group.

The DSMB charter states that there are two possible reasons for stopping the trial early, namely concerns for safety and futility. In principle, the trial will not be stopped early before the minimum number of evaluable patients required (*n* = 564) are included for beneficial effect of ibuprofen treatment on the primary outcome. The interim analyses are not associated with alpha spending.

### Statistical analysis plan

#### Overall principles

As described above the first phase of data analysis and reporting will include all outcome data up to discharge to home. Analyses will start once all data to discharge of the last included patient have been obtained, the database has been checked and locked and this SAP has been submitted for publication. The analyses will be performed by TH, WdB and RD.

Intention-to-treat analysis will be performed. Since open-label treatment might have an effect on both PDA closure and might be associated with adverse events, analyses will also be done in a per-protocol population as described in detail below. For all relevant parameters, outcome with 95% confidence intervals will be presented. A *p* value < 0.05 will be considered statistically significant. For the primary analysis, no adjustment for multiple testing will be applied. The secondary analyses are to be considered as exploratory. For statistical programming and analysis, we will use SPSS version 25.0 (IBM Corp. 2017) and the R environment for statistical computing (R Foundation for Statistical Computing, Vienna, Austria).

The short-term outcome analyses will be performed and published before the assessment of the long-term outcomes at 2 years of corrected age of all patients have been completed, since it is considered unethical by the neonatal community to withhold the results of the primary and short-term secondary outcomes for another 2 years. In the second phase, the neurodevelopmental outcomes after 2 years will be analysed, reported and published afterwards.

#### Withdrawal

As stated in the study protocol [10], the investigator or attending physician can decide to withdraw a subject from the study for urgent medical reasons. If they wish, parents or caregivers can withdraw their consent for the study at any time for any reason. Patients in the expectant management arm that meet the open-label criteria and patients in the early pharmacological treatment arm that meet the surgical ligation criteria will remain in follow up and are therefore not withdrawn from the study, as are patients that develop contraindications for continuation of ibuprofen (Fig. 2). Other reasons for withdrawal will be presented as supplementary material with reason and count per treatment group.

#### Handling of missing data

To minimize the amount of missing data, multiple attempts will be undertaken to retrieve data. Both level III and II (referral) hospitals will be contacted, since most patients will be transferred to referral hospitals after the initial hospitalization. As the primary outcome will be assessed at a PMA of 36 weeks, so generally before discharge to home, we anticipate minimal missing values. Missing data till discharge will therefore not be imputed.

For the long-term outcome at 2 years of corrected age, a higher amount of missing data is expected due to loss to follow up. In case of loss to follow-up the reason for this will be recorded. Their available trial results and characteristics will be analysed and sensitivity analyses will be performed as described later.

### Definition of analysis sets

#### Intention-to-treat population

The intention-to-treat population consists of all randomised infants, regardless of protocol deviations or use of open-label pharmacological treatment or surgical ligation (Table 3). This population includes randomised patients who died before the first ibuprofen dosage was administered.

**Table 3:** Definition of population analysis sets

Analysis population	Early pharmacological treatment group	Expectant management group
Intention-to-treat ‘as randomised’	*Early pharmacological treatment randomisation:* Including all protocol deviations.	*Expectant management randomisation:* Including all protocol deviations.
Per-protocol	*Early pharmacological treatment randomisation and treated according to study protocol:* Excluding other protocol deviations.	*Expectant management randomisation and treated according to study protocol:* Including those patients who received *‘open label’* pharmacological treatment and fulfilled the *‘open label’* criteria as described in the protcol; Excluding those patients who received *‘open label’* treatment not according to *‘open label’* criteria in study protocol; Excluding other protocol deviations.

#### Per-protocol population

In the per-protocol population, only patients included and treated in accordance with the study protocol will be included. Patients included in the expectant management group receiving pharmacological treatment after fulfilling the open-label criteria will be included in the per-protocol expectant management group. Patients in whom other protocol deviations occurred will be excluded from the per-protocol analysis (Table 3).

### Statistical analyses

#### Patient flow

**Fig. 1:**
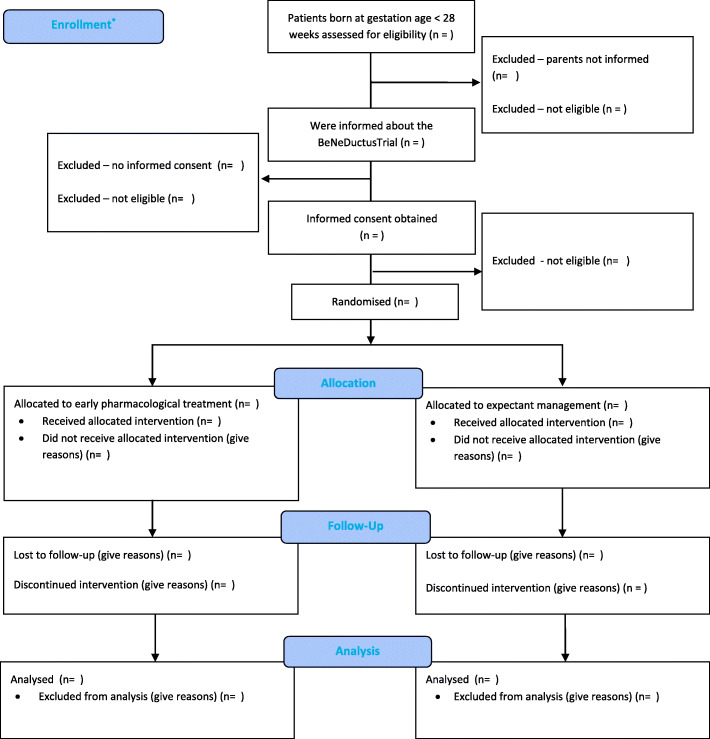
Consolidated Standards of Reporting Trials (CONSORT) 2010 flow diagram

The flow of trial participants is displayed in the CONSORT flow diagram (Fig. 1). As the echocardiographic screening for the presence of a PDA is common practice in several of the study sites, while in other centres this is considered a study procedure, patient flow differs. Therefore, informed consent is needed before an echocardiography can be performed as study procedure, while in case echocardiographic screening is common practice informed consent can be obtained after echocardiographic assessment for eligibility, as is shown in Fig.1. Reasons why patients are not eligible, and why eligible patients are not included will be summarized. Patient characteristics will be collected from all eligible infants that are not included in this study in order to assess any potential recruitment bias.

**Fig. 2:**
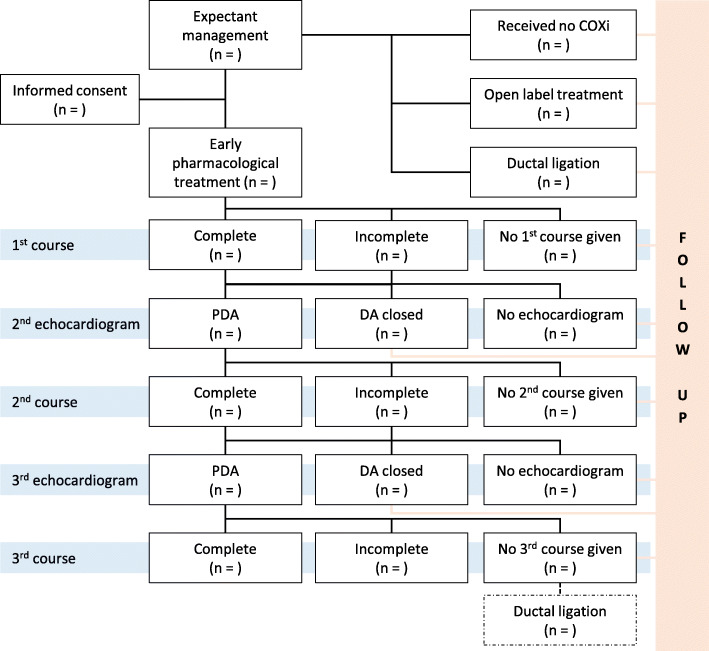
Patient flow of received treatment per treatment allocation group

The compliance to allocated treatment will be reported for all randomised patients in an additional flow chart, with reasons why allocated treatment was not given (Fig. 2).

#### Protocol deviations

Protocol deviations are defined as deviations in eligibility criteria or open-label pharmacological treatment in case the predefined criteria, as described in the study protocol, were not fulfilled. All protocol deviations will be listed as supplementary material on publication.

#### Baseline characteristics

We will present the following maternal characteristics: (a) maternal age; (b) maternal obstetrical disease, divided in (1) pre-eclampsia; (2) haemolysis, elevated liver enzymes and low platelets (HELLP) syndrome; (3) placental abruption; (4) preterm premature rupture of membranes (PPROM); and (5) clinical chorioamnionitis, defined as maternal fever without other cause or prenatal antibiotics use without other source of infection than chorioamnionitis or PPROM; (c) maternal medication, especially non-steroidal inflammatory drugs (i.e. ibuprofen and/or indomethacin) and magnesium sulphate; (d) tocolysis; (e) administration of antenatal corticosteroids, with incomplete administration defined as < 48 h or > 14 days before birth; (f) type of delivery, either vaginally or caesarean section; and (g) multiple birth (Table 1).

The following baseline characteristics of patients will be presented: (a) gestational age at birth; (b) birth weight; (c) outborn; (d) gender; (e) Apgar scores at five minutes; (f) resuscitation after birth, subdivided in (1) non-invasive respiratory support; (2) invasive respiratory support; and (3) circulatory support, defined as the need for chest compressions and/or epinephrine; (g) respiratory distress syndrome; (h) surfactant administration, and; (i) PDA diameter. All variables will be presented as summary statistics according to allocation group of the trial (Table 1).

Continuous normally distributed variables will be summarized with mean and standard deviation. Continuous non-normally distributed variables will be summarized using median and interquartile range. Categorical variables will be summarized using counts and percentages.

#### Primary outcome

Crude estimates of the relative risk and absolute risk difference of the primary outcome in the expectant management group compared with the early pharmacological treatment group will be calculated. The main analysis of the effect of expectant management on the primary outcome will be performed using 95% one sided confidence interval for the risk difference. Differences in baseline characteristics are not expected due to randomisation. In case of an unexpected disbalance in baseline characteristics correction will be made with appropriate statistical testing (i.e. logistic regression) if possible.

#### Short-term secondary outcome

Short-term secondary outcomes will be analysed using a generalized linear model. Secondary outcomes will be presented in tables with counts and percentages per treatment group and presented as absolute risk differences. No formal adjustments or multiple comparisons will be made.

#### Long-term secondary outcome at 2 years corrected age

The key long-term outcome is neurodevelopmental outcome at 2 years corrected age.

#### Subgroup analyses

We will perform pre-specified exploratory subgroup analyses for the primary outcome by examining treatment and subgroup interaction effects in logistic regression models. We will perform five analyses, each examining one subgroup: (a) gestational age groups (less than or greater or equal to 26^0/7^ weeks), (b) gender (male/female), (c) multiple pregnancy (yes/no), (d) antenatal corticosteroids (complete versus incomplete or no antenatal corticosteroids); and (e) birth weight (less than or greater or equal to 1000 g).

#### Sensitivity analyses

For BPD classification, there is uncertainty on how to categorize continuous positive airway pressure or high-flow nasal cannula with low or no supplemental oxygen [27]. The international criteria used will categorize those infants as having severe BPD [17]. In very premature infants, the reason for this support can be an impaired control of breathing rather than chronic parenchymal lung damage. Therefore, infants supported by continuous positive airway pressure or high-flow nasal cannula in room air will be classified as having mild BPD in an auxiliary sensitivity analysis in line with the STOP BPD trial [28].

If possible, we will perform sensitivity analyses on the primary outcome to investigate the impact of correlation between infant outcomes within twin or higher-order multiple births using generalized estimating equations with a logit link function.

For long-term neurodevelopmental outcome, sensitivity analyses will be performed (a) using complete cases only and by applying (b) best-case and (c) worst-case scenarios for the unobserved neurodevelopmental impairment outcome data.

#### Differences between the study protocol and statistical analysis plan

In accordance with the previously published study protocol [10], we further elaborate on the rationale of the analyses that will be performed and planned subgroup as well as sensitivity analyses.

#### Trial status

Initially, 15 participating centres started recruitment between December 2016 and April 2019, two additional centres started recruitment between August 2019 and May 2020. In another centre no patient was found to be eligible.

During the study we experienced lower inclusion rates than expected, both due to a lower rate of parental informed consent, but also a lower rate of PDA > 1.5mm than anticipated. As the funding by ZonMw stopped on 15 December 2020 and the conflicting DOXA Trial (Clinicaltrials.gov NCT04430790) started recruitment, enrollment was ended when in total 273 (48.4% of projected inclusion) patients were randomized (early pharmacological treatment n=137; expectant management n=136). The power calculation was based on data from 2008-2012, which might not represent current data. Even with the actual number of inclusions the BeNeDuctus Trial is the 3^rd^ largest RCT investigating PDA treatment since 2000 with regard to the study population. As the first two largest studies investigated prophylactic treatment [29, 30], this study is in fact the largest RCT investigating early pharmacological treatment.

The last patient is included on 5 December 2020. The last assessment of the primary outcome will be completed in March 2021. The last follow-up at 2 years corrected age is expected around March 2023. The database for the short-term outcome measurements will be locked only after data has been monitored and this SAP has been submitted for publication in a peer-reviewed journal. The long-term outcome measurements will be locked mid-2023 after monitoring of the data.

## Data Availability

The results will be presented at scientific meetings and published in peer reviewed medical journals. The data that support the findings of this study are available from the corresponding author upon reasonable request. There will be an embargo on the data for two to five years.

## Abbreviations

BPD: Bronchopulmonary dysplasia
BSID-III-NL: Bayley scales of infant and toddler development, third Dutch edition
CBCL: Child behaviour checklist
CONSORT: Consolidated standards of reporting trials
DSMB: Data safety monitoring board
IVH: Intraventricular haemorrhage
Movement ABC-2-NL: Movement assessment battery for children, second Dutch edition
NEC: Necrotizing enterocolitis
NICU(s): Neonatal intensive care unit(s)
PDA: Patent ductus arteriosus
PMA: Postmenstrual age
PNA: Postnatal age
ROP: Retinopathy of prematurity
SAP: Statistical analysis plan
TRF: Teacher report form
